# Salivary cortisol responses to acute stress vary between allergic and healthy individuals: the role of plasma oxytocin, emotion regulation strategies, reported stress and anxiety

**DOI:** 10.1080/10253890.2019.1675629

**Published:** 2019-10-24

**Authors:** L. M. Glenk, O. D. Kothgassner, A. Felnhofer, J. Gotovina, C. L. Pranger, A. N. Jensen, N. Mothes-Luksch, A. Goreis, R. Palme, E. Jensen-Jarolim

**Affiliations:** a Comparative Medicine, The Interuniversity Messerli Research Institute of the University of Veterinary Medicine Vienna, Medical University Vienna and University Vienna, Vienna, Austria;; b Department of Child and Adolescent Psychiatry, Medical University of Vienna; Division of Clinical Psychology at the Medical Directorate of the Vienna General Hospital, Vienna Medical Campus, Vienna, Austria;; c Department of Child and Adolescent Medicine, Medical University Vienna, Austria;; d Institute of Pathophysiology and Allergy Research, Center of Pathophysiology, Infectiology and Immunology, Medical University of Vienna, Vienna, Austria;; e AllergyCare, Allergy Diagnosis and Study Center, Vienna, Austria;; f Department of Applied Psychology: Health, Development, Enhancement and Intervention, Faculty of Psychology, University of Vienna, Austria;; g Outpatient Unit for Research, Teaching and Practice, Faculty of Psychology, University of Vienna, Vienna, Austria;; h Unit of Physiology, Pathophysiology und experimental Endocrinology, University of Veterinary Medicine Vienna, Vienna, Austria

**Keywords:** Allergy, cortisol, stress, emotion regulation, oxytocin, emotion suppression, reappraisal

## Abstract

Numerous studies have demonstrated that acute psychological stress, induced by the Trier Social Stress Test (TSST) paradigm, affects salivary cortisol secretion and self-reported stress measures including anxiety. Allergy has been related to altered cortisol responsiveness and increased stress vulnerability. Here, we investigated acute stress responses and emotion regulation strategies in cohorts of allergic and healthy individuals. Groups of allergics and healthy individuals were subjected to the TSST and experienced levels of stress and anxiety, as well as emotion regulation strategies, were assessed. Cortisol and oxytocin concentrations were measured in saliva or plasma. The present findings confirm earlier results of altered stress responsiveness in allergic individuals. Acute stress by the TSST evoked higher physiological arousal in allergics by means of salivary cortisol secretion. Allergics also scored higher on emotion suppression. However, individuals who were more likely to use reappraisal recovered more efficiently from the cortisol increase. No such effect for reappraisal was found in the healthy population. No differences in self-reported anxiety and stress emerged between the groups. Plasma oxytocin levels prior to the TSST were significantly higher in allergics. Our data corroborate earlier findings on altered stress susceptibility in allergics. Moreover, we identified differences in emotion regulation and oxytocin secretion which should be further explored. Accounting for the emerging global prevalence of allergy, more in-depth research into the experience of stress, coping strategies and stress-related molecules in allergic people is warranted.

**Short summary**
This study addressed stress experiences and emotion regulation in allergic and non-allergic adults. Allergics scored higher on emotion suppression, had higher pre-stress concentrations of plasma oxytocin and exhibited a stronger salivary cortisol response to stress than healthy people. The research outcomes indicate that allergic individuals cope less efficiently with acute stress but may benefit from adaptive emotion regulation strategies such as reappraisal.

## 1. Introduction

Psychosocial stress is a complex phenomenon with an emerging prevalence in modern societies. Stress responsiveness has been identified as a crucial factor associated with the onset and progression of diseases including allergy (Dave, Xiang, Rehm, & Marshall, 2011; Montoro et al., 2009). In immediate type allergy, immunoglobulins E (IgE) specific for an allergen bind with high affinity to IgE receptors expressed on inflammatory cells. Upon subsequent encounter, the allergen triggers via IgE the immediate release of inflammatory mediators such as histamine. The appropriate responsiveness of the hypothalamus-pituitary adrenal (HPA) axis has been considered essential in the control of inflammation and in the prevention of an overshooting immune response (Hänsel, Hong, Cámara, & von Känel, 2010) like allergy. Disrupted or blunted HPA axis activity has been suspected to play a role in the etiological process of allergies (Stenius et al., 2011). Research in psycho-allergology has revealed that allergics are more vulnerable to stress and more prone to anxiety disorders (Buske-Kirschbaum, Ebrecht, Kern, Gierens, & Hellhammer, 2008). Emotion regulation, the ability by which an individual may influence the quality, perception and expression of an emotion (Gross, 2002), modulates physiological reactivity. According to Wang and Saudino (2011), stress experiences and emotion regulation are characterized by a high individual variability and shared neural mechanisms. Two distinct patterns of emotion regulation have been described by Gross (1998), previously mentioned by Carver, Scheier, and Weintraub (1989) as coping with stress strategies. Emotion suppression relates to a response-focused strategy to block an ongoing emotional perception. In contrast, reappraisal is an antecedent-focused approach including any attempt or behavior used to reframe or reevaluate a situation before an emotion is manifest. As demonstrated by Lam, Dickerson, Zoccola, and Zaldivar (2009), higher cortisol reactivity to a social-evaluative speech task was related to high levels of both emotion suppression and reappraisal in healthy individuals. However, to our knowledge, trait emotion regulation strategies with regard to acute stress have not yet been studied in an allergic population. Determination of oxytocin with regard to its stress-buffering function has received significant attention over the past decade. Oxytocin is a neuromodulator that has been extensively studied and exerts a wide array of psychobiological functions. Oxytocin is a potent stress mediator as it downregulates the HPA axis response during social stress experiences (Heinrichs, Baumgartner, Kirschbaum, & Ehlert, 2003) and reducing autonomic nervous system arousal with considerable effects on the behavioral output of an organism (Uvnäs-Moberg & Petersson, 2005). In fact, oxytocin may dampen stress responses at the interface of physiological arousal and emotional processing as has been recently reported by Koch et al. (2019). From an immunological view it is worthy of mention that mast cells express oxytocin receptors and may thus connect this psychosocial mediator also with immune responses in allergy (Vega & Rudolph, 2002). The Trier Social Stress Test (TSST) is a standardized laboratory protocol to induce moderate psychobiological arousal (Kirschbaum, Pirke, & Hellhammer, 1993). The goal of this exploratory study was to examine whether allergic individuals, when compared to a cohort of healthy controls, differ in their emotion regulation strategies, self-reported anxiety and stress in conjunction with HPA axis responsiveness and peripheral oxytocin levels in response to acute stress. We hypothesized that allergics would be more likely to exhibit an inefficient HPA axis response to the stressful experience, evident in either a dampened cortisol response or a prolonged recovery period. In addition, we also expected to measure higher levels of self-reported stress and anxiety in the allergic cohort. Moreover, we hypothesized that emotion regulation strategies and oxytocin secretion may account for potential differences in cortisol responsiveness.

## 2. Material and methods

### 2.1. Participants

Study volunteers were recruited through advertisement at the University of Veterinary Medicine Vienna, the Medical University Vienna and the University of Vienna and selected via an online screening questionnaire, resulting in 40 age and gender matched study participants (21 males and 19 females; 21–34 years) who met the inclusion criteria. As demonstrated in Table 1, these persons were allocated to (a) an allergic patients group (*n* = 19), who reported typical seasonal or perennial clinical symptoms as well as doctor-diagnosed allergies to grass pollen, birch pollen and/or house dust mites and (b) a healthy control group (*n* = 21), characterized by the absence of clinical allergy symptoms and without doctor-diagnosed allergies. None of the allergic patients had symptoms other than mild-to-moderate allergic rhinitis at the time recruited, with some reporting mild oral allergy syndrome to apple due to IgE crossreactivity with birch pollen. Prestudy screening of volunteers via skin prick testing (SPT) and determination of IgE levels are reported elsewhere (Gotovina et al., 2018). In the allergic cohort, polysensitization was prevalent in 15 out of 18 participants. More specifically, eight individuals had positive SPT reactions for the three allergens birch pollen (BP)/grass pollen (GP)/house dust mite (HDM); three individuals had positive SPT reactions for BP/GP; two individuals had positive SPT reactions for BP/HDM or GP/HDM, respectively. All control participants declared to be medication free and not to suffer from an acute or chronic disease. Female participants reported not to use any hormonal contraceptives and were tested in the luteal phase of their menstrual cycle to control for cycle-dependent endocrine stress reactivity (Kirschbaum, Kudielka, Gaab, Schommer, & Hellhammer, 1999; Villada et al., 2014). The cycle phase was determined during prestudy interviews with female study volunteers and experiments were arranged accordingly. We used this schedule accounting for the results by Kirschbaum et al. (1999), who reported no differences in cortisol levels between women during the luteal cycle phase and men. Also, the age range of participants admitted to this study was between 20–35 years to control for age-specific endocrine variations (Kudielka, Schommer, Hellhammer, & Kirschbaum, 2004). Considering the effects of circadian cortisol rhythmicity, experiments were only scheduled in the early afternoon in mid flowering season between May and June. None of the allergic participants had received treatment with steroids, antihistamines or inhalant over the past 12 weeks before recruitment and before the test. None of the control participants had ever suffered from allergy. Only volunteers who were nonsmokers and agreed not to consume alcohol or caffeine on the day of the experiment and not to eat for at least ninety minutes before the actual start of the experimental protocol were considered for participation. One allergic participant signed the informed consent but refused to provide blood samples during the study protocol and was therefore not considered in the analysis. Thus, a total of *N* = 18 allergic persons and *N* = 21 nonallergic healthy persons aged from 21 to 34 years (Mean = 25.08, SD = 3.047) participated in the study. The study protocol was approved by the ethics committee of the Medical University of Vienna (EK Nr 1030/2015). The study was carried out in AllergyCare®, Allergy Diagnosis and Study Center Vienna.

**Table 1.** Characterization and clinical description of study participants.

Descriptors	Allergic (number)	Healthy (number)
Sample	18 (46.15%)	21 (53.85%)
Gender distribution	9 ♂	10 ♂
	9 ♀	11 ♀
Asthma	4	–
Total IgE > 100 IU/ml	10	1
Doctor-diagnosed allergies	18	–
*Grass pollen*	8	–
*Birch pollen*	13	–
*House dust mite*	12	–
Additional other allergens	10	–
Allergic rhinitis symptoms^a^	6	–
Self-reported perception of symptom severity^b^	5	–

a Patients reporting to currently suffer from at least 3 typical rhinitis symptoms (sneezing, nasal pruritus, airflow obstruction or nasal discharge; Wheatley & Togias, 2015).

b Numbers of patients with a self-reported perception of current allergic rhinitis symptoms > 3, on a likert-type scale ranging from 1 (minimal) to 5 (maximal symptom level).

### 2.2. Psychometric measures

State anxiety was assessed before and after the TSST using the German state subscale of the *State-Trait-Anxiety-Inventory (STAI-S*, Laux & Spielberger, 2001). The STAI-S is a 20-item self-report measure (4-point Likert scale) which has previously been extensively used in stress research. Similar to state anxiety, emotion regulation was measured prior to and after the TSST with the German version of the *Emotion Regulation Questionnaire (ERQ*; Abler & Kessler, 2009). It consists of 10 items which assess the two common emotion regulation strategies *Reappraisal* and *Suppression*. While the former comprises cognitive strategies to decrease a situation’s emotional impact, the latter consists of inhibiting the expression of feelings (Gross, 2002). Finally, participants were asked to indicate their perceived stress level (“How stressed are you?”) on a 10 cm *Visual Analog Scale (VAS)* at four time points during the experiment (see study protocol below).

### 2.3. Saliva sampling

Commercial sampling devices (Salivette®, 51.1534, Sarstedt, Wiener Neudorf, Austria) without any saliva-stimulating additives were used to obtain saliva. After signing the informed consent, study participants were instructed how to collect their saliva via putting the cotton roll from the sampling tube into their cheek pouch, letting it saturate with saliva for approximately 60–80 seconds and finally replacing it in the device container. Sampling tubes were immediately stored in a freezer at −20 °C in the laboratory facility at the research site.

### 2.4. Blood sampling

Blood samples were drawn from the right arm before the stress test and from the left arm after the test and stored in EDTA containing vials (Vacutainer^®^). The samples were centrifuged for 10 min at 3000×*g* at room temperature and plasma samples were collected in Eppendorf vials and immediately stored in a freezer at −80 °C in the laboratory facility until further analyses were done.

### 2.5. Study protocol

**Figure 1.**
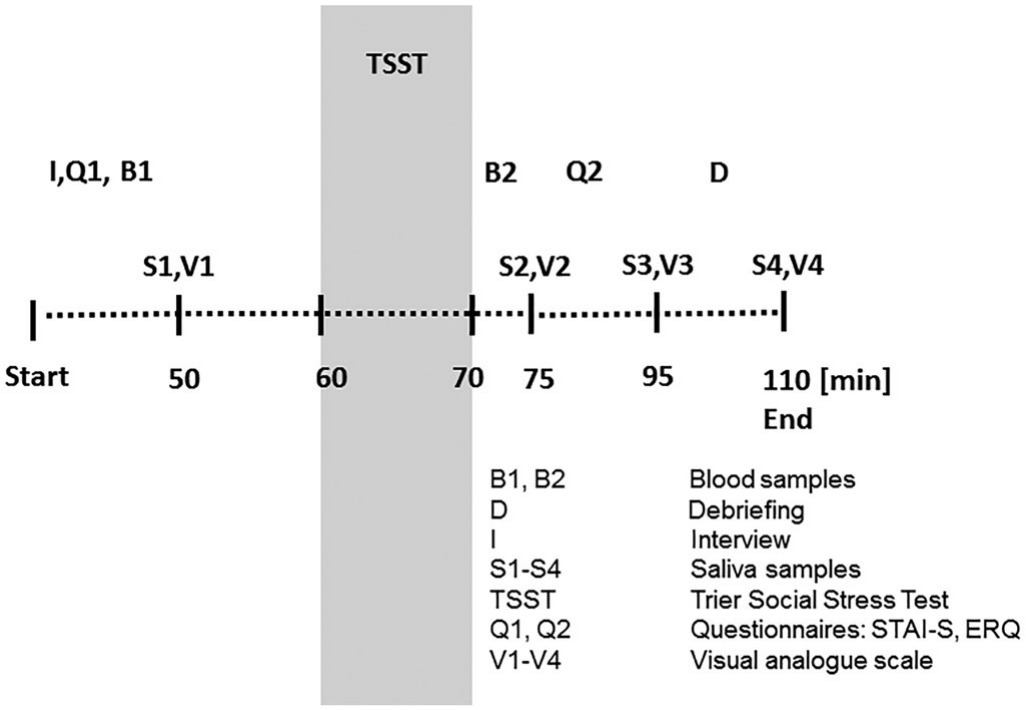
Schematic overview of the study design and procedures.

Upon arrival, individuals were provided with information sheets and written informed consent was obtained. As indicated in Figure 1, demographic data including age, gender and educational background, as well as individual medical history, were assessed during an interview (I) with a study assistant. Afterwards, the psychometric questionnaires STAI-S and ERQ were completed (Q1) and experienced stress was rated on a visual analog scale (V1). Simultaneously, a saliva sample (S1) was collected to obtain a baseline value after participants had sufficient time to settle and adapt to the environment. After 60 minutes, participants were guided to the experimental room by the study assistant and completed the TSST. The experimental protocol included an anticipation period (5 minutes), where the recipients were required to prepare a free speech, followed by the actual TSST (10 minutes) in front of a committee. During the latter, recipients had to deliver their speech (5 min) and perform a spontaneous mental arithmetic task (5 min). The members of the committee gave only short instructions and did not provide any verbal or nonverbal support or reassurance to the recipients throughout the exposition. Postconfrontation measures (S2, V2) were assessed five minutes after completion of the TSST. During the next 20 minutes, the second STAI-S questionnaire (Q2) was administered and participants collected their saliva and rated experienced stress (S3, V3). During the debriefing process (D), participants were provided with an informative explanation of the hypothesis being tested and the methods used. Consecutively, the final data (S4, V4) were gathered.

### 2.6. Salivary cortisol analysis

Prior to analysis, samples were thawed on ice and centrifuged at room temperature at 3000×*g* for 15 min to obtain saliva. Aliquots (10 μl of a 1:10 dilution) of clear saliva were used for the analysis of cortisol. Analyses were carried out at the University of Veterinary Medicine in Vienna using a highly sensitive enzyme immunoassay kit. Samples were assayed in duplicates and cortisol concentrations were assessed in a double-antibody biotin-linked enzyme immunoassay (sensitivity of 0.2 ng/ml; Palme & Möstl, 1997). Duplicate samples with a coefficient of variation >10% were replicated and considered in the analysis only when a coefficient of variation <10% was achieved. If sample volumes were below the limit needed to run duplicates or sample volumes were too low before a coefficient of variation <10% was achieved, the sample was dismissed. Average intra- and inter-assay coefficients of variation were less than 10% and 13%, respectively.

### 2.7. Plasma oxytocin analysis

Prior to analysis, samples were thawed on ice and centrifuged at room temperature at 1000×*g* for a few minutes. Plasma levels of oxytocin (detection limit 15 pg/ml) were determined using enzyme-linked immunoassay (ELISA) according to the manufacturer's recommendations (Enzo, Lausen, Switzerland). Concentrations that varied by more than 5% were subject to repeat testing or dismissed if sample volumes were too low before a coefficient of variation <5% was achieved.

### 2.8. Data analysis plan

Repeated measures ANOVAS were computed to analyze differences between allergic and non-allergic participants in self-reported measures of stress and state anxiety. Sex was not a significant covariate in any of our analyses; it was therefore omitted from further models. We analyzed the cortisol response to the TSST in our sample with piecewise multilevel modeling (MLM) to analyze differences in cortisol reactivity, and cortisol recovery. All statistical analyses were conducted using R version 3.5.0 (R Core Team, 2018). Differences between the allergic and healthy samples were analyzed using t-tests between cortisol and oxytocin samples, subscales of emotion regulation, and trait anxiety. Multilevel models were fitted using the lme4 package (Bates, Mächler, Bolker, & Walker, 2015) with *p*-values supplied by the lmer package (Kuznetsova, Brockhoff, & Christensen, 2017). Cortisol concentrations were log-transformed to reduce skewness. To examine within- and between-person change in cortisol responses simultaneously, we specified within-person change in level 1 to examine cortisol responses to the TSST and a between-person submodel in level 2, describing how cortisol responses vary between the allergic and non-allergic groups. We examined instantaneous change (time) and quadratic change trajectories (time^2^) for cortisol levels in response to the TSST. Power analysis using G*Power (Faul, Erdfelder, Lang, & Buchner, 2007) revealed that our study had a power of .97 to find a medium effect size (i.e. interaction) in our repeated measures ANOVAS. The sample size of 39 is within the recommended range of level 2 grouping variables to establish accurate estimates in multilevel-modeling (Maas & Hox, 2005).

Two models were fit. Our first model included change trajectory slopes and their cross-level interaction with allergy status (0 = nonallergic, 1 allergic). Our second model additionally included changes in oxytocin concentration (from −10 min pre-TSST to immediately after the TSST), trait anxiety and ERQ suppression/reappraisal with both change trajectory slopes and allergy status. ERQ subscales, changes in oxytocin concentration, and trait anxiety variables were standardized to have means of 0 and standard deviations of 1. A preliminary unconditional model revealed that sex was not significantly associated (*p*=.852) with changes in cortisol levels, we therefore did not include sex in our models.

Model 1:

Level 1

$$\mathrm{Cortisol}\;(\log\;ng/ml)=\;\beta_{0j}+\;\beta_{1j}t_{1ij}+\beta_{2j}t_{1ij}^{2}+R$$

Level 2

$$\beta_{0j}=\;\gamma_{00}+\gamma_{01}{\mathrm{Allergy}}_{j}+\upsilon_{0j}$$

$$\beta_{1j}=\;\gamma_{10}+\gamma_{11}{\mathrm{Allergy}}_{j}+\upsilon_{1j}$$

$$\beta_{2j}=\;\gamma_{20}+\gamma_{21}{\mathrm{Allergy}}_{j}+\upsilon_{2j}$$

Model 2:

Level 1

$$\mathrm{Cortisol}\;(\log\;ng/ml)=\;\beta_{0j}+\;\beta_{1j}t_{1ij}+\beta_{2j}t_{1ij}^{2}+R$$

Level 2

$$\beta_{0j}=\;\gamma_{00}+\gamma_{01}{\mathrm{Allergy}}_{j}+\gamma_{02}{\mathrm{OxyDelta}}_{j}+\gamma_{03}{\mathrm{Anxiety}}_{j}+\gamma_{04}{{\mathrm{ERQ}}_{\mathrm{Reappraisal}}}_{j}+\gamma_{05}{{\mathrm{ERQ}}_{\mathrm{Supression}}}_{j}+\upsilon_{0j}$$

$$\beta_{1j}=\;\gamma_{10}+\gamma_{11}{\mathrm{Allergy}}_{j}+\gamma_{12}{\mathrm{OxyDelta}}_{j}+\gamma_{13}{\mathrm{Anxiety}}_{j}+\gamma_{14}{{\mathrm{ERQ}}_{\mathrm{Reappraisal}}}_{j}+\gamma_{15}{{\mathrm{ERQ}}_{\mathrm{Supression}}}_{j}+\upsilon_{1j}$$

$$\beta_{2j}=\;\gamma_{20}+\gamma_{21}{\mathrm{Allergy}}_{j}+\gamma_{22}{\mathrm{OxyDelta}}_{j}+\gamma_{23}{\mathrm{Anxiety}}_{j}+\gamma_{24}{{\mathrm{ERQ}}_{\mathrm{Reappraisal}}}_{j}+\gamma_{25}{{\mathrm{ERQ}}_{\mathrm{Supression}}}_{j}+\upsilon_{2j}$$

## 3. Results

Table 2 presents the means and standard deviations of all study variables for allergics and non-allergic individuals. Emotion regulation strategies (ERQ) were assessed in allergic and non-allergic individuals (Table 2). Group comparisons indicate significant group differences in *suppression* strategies (*t*(37)=2.761, *p*=.009, *d* = 0.89) with allergic participants reporting to use suppression of emotions more frequently (*M* = 18.17, *SD* = 4.10) than their nonallergic counterparts (*M* = 14.29, *SD* = 4.59). Regarding the emotion regulation strategy *reappraisal*, however, there were no significant group differences (*t*(37)= −1.015, *p* = .316).

**Table 2.** Descriptive statistics for allergics and nonallergics in the current sample.

	Allergics (*n* = 18)	Nonallergics (*n* = 21)	χ^2^
	*n* %	*n* %	
Sex			0.022
Male	9 (50)	10 (48)	
Female	9 (50)	11 (52)	
	*M (SD)*	*M (SD)*	*t*
Age	25.33 (3.61)	24.86 (2.54)	0.482
Oxytocin S1 (pg/ml)	1187.62 (256.59)	997.74 (230.65)	2.335*
Oxytocin S2 (pg/ml)	1118.42 (251.76)	1027.50 (236.26)	1.117
Δ Oxytocin (pg/ml)	−69.21 (124.17)	29.76 (189.52)	1.853
Cortisol S1 (log ng/ml)	2.59 (0.62)	(2.69) (0.49)	−0.555
Cortisol S2 (log ng/ml)	2.83 (0.61)	2.72 (0.59)	0.589
Cortisol S3 (log ng/ml)	2.95 (0.52)	2.65 (0.60)	1.658
Cortisol S4 (log ng/ml)	2.73 (0.48)	2.63 (0.59)	0.535
Reappraisal	15.39 (8.42)	18.38 (9.77)	1.015
Suppression	18.17 (4.11)	14.29 (4.60)	2.761**
Trait Anxiety	36.56 (9.83)	35.19 (7.36)	0.495

*p<.05; **p<.01; ***p<.001.

### 3.1 Multilevel models predicting change in cortisol responses

**Figure 2.**
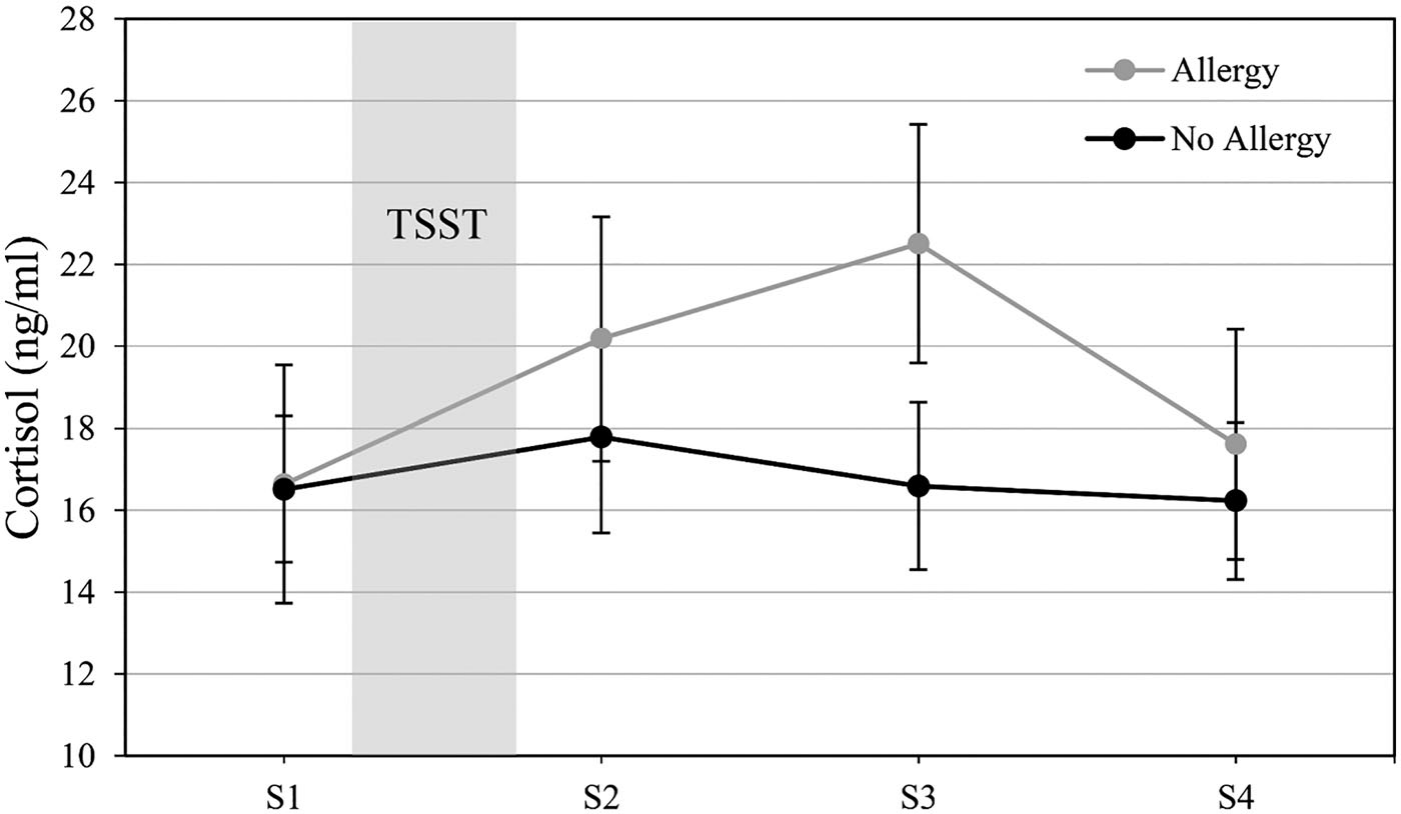
Salivary cortisol (*M*±*SEM*) responses at predefined time points before (S1) and after (S2–S4) the Trier Social Stress Test (TSST) in non-allergic and allergic study participants.

Cortisol levels for the allergy and nonallergy groups are presented in Figure 2 (raw cortisol levels are depicted in ng/ml for comparability). Analysis without predictors confirmed that cortisol levels were increasing at the start of the TSST (*b* = 0.182, *SE* = 0.062, *p*=.004) and the cortisol response can be characterized by a quadratic change trajectory (*b*= −0.057, *SE* = 0.019, *p*=.004). Our first model with allergy status indicated a significant interaction of allergy*time (*b* = 0.379, SE = 0.120, *p*=.002), indicating a steeper rise in cortisol levels for allergics. The interaction between allergy and time^2^ (i.e. curvature) was significant as well (*b*= −0.101, *SE* = 0.037, *p*=.008) with allergics displaying a less steep recovery slope than nonallergics.

In model 2, reappraisal showed a significant interaction with allergy, and quadratic change (*b* = 0.075, *SE* = 0.033, *p*=.026). Simple slope analysis revealed that this association between the acceleration of cortisol levels over time (i.e. steeper recovery slope) and reappraisal was found in allergics (*b* = 0.078, *SE* = 0.037, *p*=.043) but not in non-allergics (*b* = 0.008, *SE* = 0.024, *p*=.775). The remaining interactions tested in model did not reach significance. Model summaries are depicted in Table 3.

**Table 3.** Model summaries of multilevel models.

	Model 1		Model 2	
	*b (SE)*	*p*	*b (SE)*	*p*
(Intercept)	2.699 (0.124)	<.001	2.632 (0.129)	<.001
Time	0.008 (0.081)	.919	0.064 (0.087(	.465
Time^2^	−0.011 (0.025)	.668	−0.023 (0.027)	.387
Allergy	−0.119 (0.182)	.518	−0.053 (0.182)	.774
Time*Allergy	0.379 (0.120)	.002	0.264 (0.135)	.053
Time^2^*Allergy	−0.101 (0.037)	.008	−0.061 (0.042)	.148
Time*Allergy*OxyDelta			−0.035 (0.133)	.794
Time^2^*Allergy*OxyDelta			0.035 (0.042)	.410
Time*Allergy*Reappraisal			−0.188 (0.102)	.070
Time^2^*Allergy*Reappraisal			0.075 (0.033)	.026
Time*Allergy*Suppression			0.051 (0.128)	.692
Time^2^*Allergy*Suppression			−0.014 (0.040)	.735
Time*Allergy*Anxiety			−0.141 (0.090)	.123
Time^2^*Allergy*Anxiety			0.057 (0.029)	.052

### 3.2 Plasma oxytocin

Oxytocin was assessed as an additional physiological parameter before and after the TSST via blood sampling and as depicted in Table 2, oxytocin levels prior to the TSST were significantly higher in allergics (*M* = 1187.62, *SD* = 256.59 pg/ml) than in nonallergics (*M* = 997.74, *SD* = 230.65 pg/ml; *t*(34)=2.335, *p*=.077, *d*= −0.651). Post-TSST oxytocin values of allergics (*M* = 1118.42, *SD* = 251.76 pg/ml) and nonallergics (*M* = 1027.50, *SD* = 236.26 pg/ml) did not differ significantly (*t*(34)=1.117, *p*=.272). In addition, differences in oxytocin concentrations (Delta oxytocin) were calculated at the time points B1 minus B2. Delta oxytocin exhibited a trend to significant differences suggesting that the stress experience led to decreases in oxytocin levels in allergic participants, while in healthy controls, concentrations of the neuropeptide hormone increased (*t*(34)=1.853, *p*=.073, *d* = 0.62).

### 3.3 Experienced stress and anxiety measures

**Figure 3.**
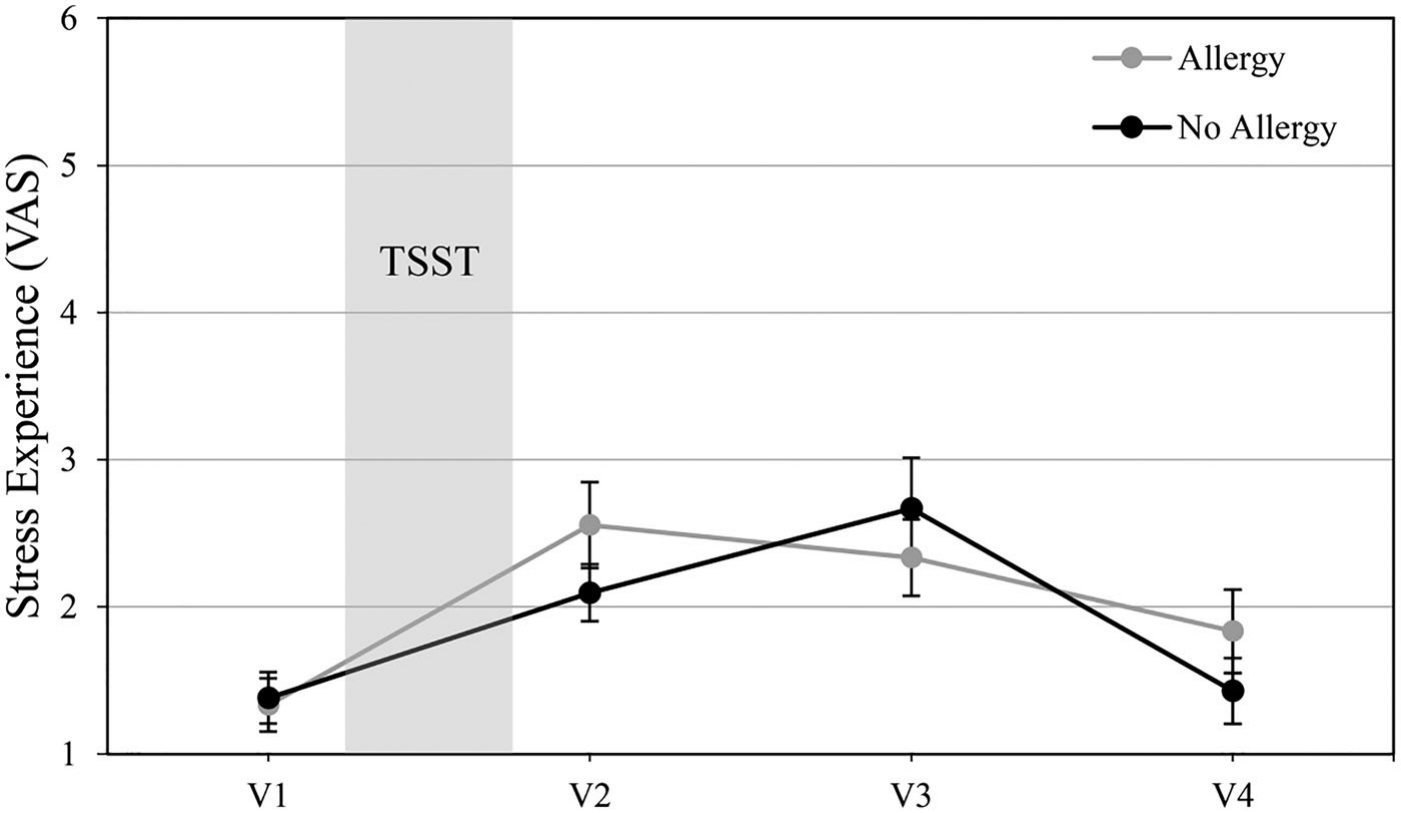
Visual analog scale measures of experienced stress (*M*±*SEM*) before (V1) and after (V2–V4) the Trier Social Stress Test (TSST) in non-allergic and allergic study participants. V1 was assessed after arrival at the research facility, V2 was assessed after the TSST, V3 and V4 refer to post-stress measures following a recovery period.

**Figure 4.**
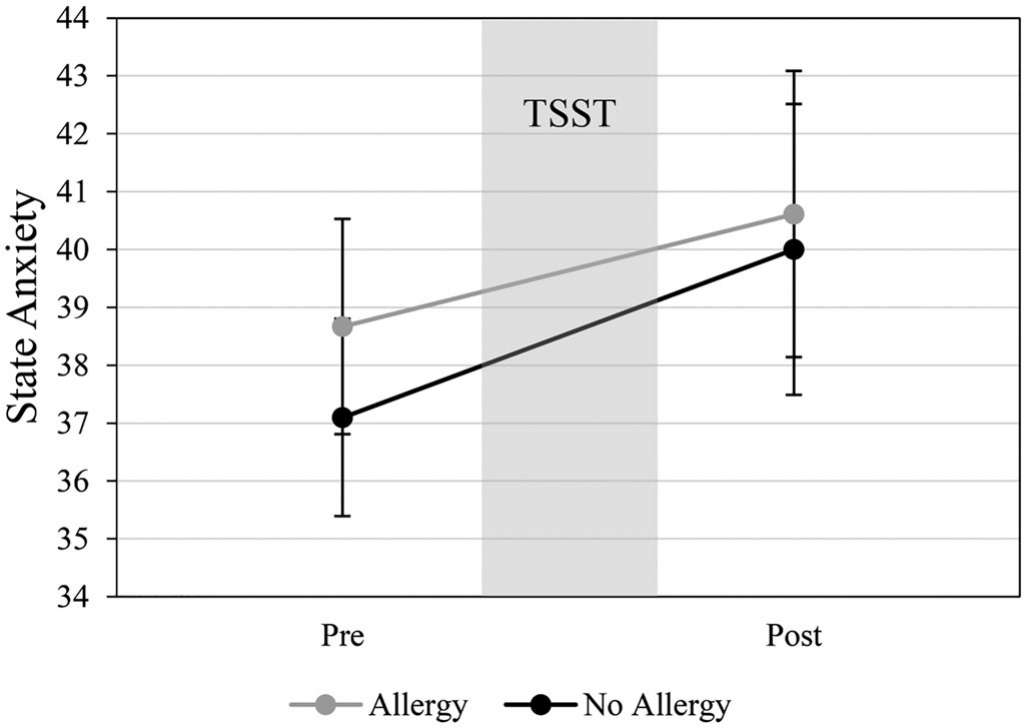
State Anxiety measures (M ± SEM) before (Q1) and after (Q2) Trier Social Stress Test (TSST) in non-allergic and allergic study participants.

The VAS stress ratings over 4 time points (−10, +5, +25, and +40) showed a significant main effect over time (*F*(3, 111)=15.498, *p*<.001, par. η^2^=0.295), but no significant interaction of VAS ratings*allergic status was found (*F*(3, 111)=1.860, *p*=.140, par. η^2^=0.048). State anxiety measures before and after stress exposure only revealed higher STAI ratings after the TSST procedure (*F*(1, 37)=5.177, *p*=.029, par. η^2^=0.123), but no interaction effect of STAI x allergic status groups (*F*(1, 37)=0.203, *p*=.655, par. η^2^=0.005). See Figures 3 and 4 for a detailed depiction of experienced stress and state anxiety responses.

## 4. Discussion

According to the World Allergy Organization (WAO), recent evidence about the increasing prevalence of allergic diseases worldwide warrants extensive research into the subject (Pawankar, Canonica, Holgate, Lockey, & Blaiss, 2013). The European Academy of Allergy and Clinical Immunology (EAACI) estimates that by 2025, more than half of the European population will suffer from some type of allergy (EAACI, 2016). Thus, allergy has been recognized as a global public health issue. From the healthcare perspective, the burden of allergic diseases on quality of life determinants is notably important as the degree of the severity of allergic symptoms impacts psychological well-being that, in turn, modulates social interaction (Pawankar et al., 2013).

*Prima facie*, our findings reveal that both allergics and nonallergics responded to the TSST on the level of salivary cortisol secretion and self-reported anxiety and stress. However, in comparison to the self-reported stress measures, only cortisol responses differed significantly between the two groups. Interestingly, allergic individuals exhibited higher HPA axis responses by means of cortisol increases than non-allergic individuals. These outcomes are particularly interesting in light of existing research pointing at blunted cortisol responses during acute stress generated by the TSST in individuals with chronic allergy (Buske-Kirschbaum, Geiben, Hollig, Morschhauser, & Hellhammer, 2002, Buske-Kirschbaum, Geiben, Hollig, & Hellhammer 2002). On the other hand, previous results on a cohort of men and women with allergic rhinitis subjected to the TSST also demonstrated a substantial increase in cortisol levels (Kiecolt-Glaser et al., 2009). Our results on a cohort of mainly seasonal allergics indicate that allergic individuals may even respond stronger on the level of cortisol secretion than healthy controls during an acute stress scenario. This is not unexpected as previous research has pointed toward an increased vulnerability for stress-related anxiety in allergic populations (Buske-Kirschbaum et al., 2008).

Apparently, in contrast to our hypothesis, the study findings revealed heightened stress responsiveness in allergics only on the level of physiological arousal, not in any of the self-report measures (STAI-S or the visual analog scale). The discrepancy between self-reported stress levels and physiological arousal may be interpreted as an effect of interfering emotion regulation strategies (Gross & Levenson, 1993) and/or individual attribution styles.

Our data support earlier results by Lam et al. (2009) who found that individuals who scored higher on emotion suppression also had stronger cortisol increases in response to acute stress. In our sample, allergic individuals exhibited higher scores in emotion suppression and a steeper increase in salivary cortisol secretion. In light of stressful experiences, it is notable that the inhibition of overt emotion expression has been linked to lower social support, less closeness to others, and lower social satisfaction (Srivastava et al. 2009). Moreover, negative mood has been associated with higher emotion suppression during stressful interactions in interpersonal relationships (Côté, Gagnon-Girouard, Sabourin, & Bégin, 2018).

However, allergic individuals recovered more efficiently from the stress-induced cortisol rise if they used reappraisal. This association of appraisal on the HPA stress cascade is in line with results from Jamieson et al. (2012), who reported that cardiovascular stress responses were more efficient in individuals using appraisal. In contrast to emotion suppression, reappraisal is an adaptive strategy to attenuate physiological reactivity to stress and negative affective experiences (Jamieson, Mendes, & Nock, 2013). Oxytocin has been attributed a significant role in facilitating complex social behaviors and in modulating neural circuits for social interactions (Marlin & Froemke, 2017). Determination of plasma oxytocin revealed a significant difference in prestress concentrations in the allergic cohort. Higher baseline oxytocin has previously been related to increased anxiety and relationship distress (Taylor et al., 2006; Turner, Altemus, Enos, Cooper, & McGuinness, 1999). No effect of oxytocin on the cortisol reaction was found, which parallels earlier findings by Taylor et al. (2006) but contrasts research on adolescents (Bernhard et al., 2018). Our descriptive data indicate that the TSST provocation resulted in reduced oxytocin concentrations in allergic participants, while in their nonallergic counterparts oxytocin increased, marked by a trend toward significance in delta oxytocin. This is interesting as oxytocin acts on subcortical regions like the amygdala and associated systems, which are related to the formation of anxiety (Kirsch et al., 2005). In sum, the present results again reflect altered stress coping strategies in allergic individuals. However, a replication of the experiment with a lager sample size would be essential. In sum, future research is challenged to further debunk associations between emotion regulation, physiological stress reactivity and self-reported stress responses.

A limitation of our study is that in the context of the pilot nature of the study, according to the relatively small sample size, we were not able to control for seasonality and chronicity of allergy. Controlling for these factors would be worthwhile in light of results by Buske-Kirschbaum, Ebrecht, and Hellhammer (2010) who demonstrated that in seasonal allergy, attenuated HPA axis responsiveness to psychosocial stress occurs exclusively during the acute inflammatory state while the phenomenon is absent when participants were tested during the non-symptomatic period. In the present study, gender was balanced in both cohorts and according to the analysis, no effect of gender on the difference on the cortisol responses, emotion regulation or self-reported stress was prevalent. However, taken the significant role of gender in allergy into account, future research will also need to consider gender differences in the psychobiological and allergy-related mechanisms that shape the individual stress response. Although salivary cortisol is the most frequently measured biomarker in laboratory stress research because it represents the unbound and thus, biologically active amount of cortisol and is independent of the saliva flow rate (Kirschbaum & Hellhammer, 2000), additional stress-related molecules should be considered. There is evidence that plasma and salivary oxytocin are correlated (Grewen, Davenport, & Light, 2010). Despite initial criticism towards the determination of oxytocin in saliva (Horvat-Gordon, Granger, Schwartz, Nelson, & Kivlighan, 2005), recent data indeed suggest that salivary oxytocin may, in fact, better reflect cerebrospinal fluid concentrations than plasma levels and should, therefore, be used in future studies (Martin et al., 2018). Of note, there is some evidence that extraction prior to analysis of oxytocin with commercial assays is likely to increase data validity by lessening the amount of assay cross-reactivity (McCullough, Churchland, & Mendez, 2013). Thus, addressing this study’s limitations, a replication of the experiment with an increased sample size and update of the study protocol would be worthwhile. As psychosocial stress activates an array of neuroendocrine and immunological responses, it would be especially interesting to combine the study of salivary protein release, plasma mediators and leukocyte populations with psychometric variables like emotion regulation strategies that, according to the present findings, compile an important component of individual coping with stress in allergic populations.
